# Complex Coronary Interventions: Are We Reaching a Plateau?

**DOI:** 10.3390/jpm12081298

**Published:** 2022-08-08

**Authors:** Luca Franchin, Giacomo Boccuzzi, Diego Moniaci, Mario Iannaccone

**Affiliations:** 1 Division of Cardiology, Department of Medicine, San Giovanni Bosco Hospital, ASL Città di Torino, 10100 Turin, Italy; 2 Division of Vascular Surgery, Department of Surgery, San Giovanni Bosco Hospital, ASL Città di Torino, 10100 Turin, Italy

The complexity of coronary artery disease is currently on the rise [1]. As a result, the field of interventional cardiology has expanded rapidly in the last years to keep up with the necessity of treating more complex anatomies and patients. To answer this need, novel techniques and equipment have been developed to face the new challenges of coronary intervention. Complex high-risk and indicated percutaneous coronary intervention (CHIP) is an evolving concept that englobes many factors such as procedural aspects and hemodynamic status that must be taken into consideration before percutaneous coronary intervention (PCI). However, a substantial comorbidity burden, including frailty and oncologic diseases, affects the aging population, so risk stratification remains difficult. Most of these patients have been excluded from RCTs whereas observational studies only include patients who underwent revascularization [2]. Despite the lack of a clear definition of CHIP it usually involves left main PCI, calcium atherectomy, chronic total occlusions, and left ventricular support assisted PCI. To date, reaching the best clinical outcomes requires clear and straightforward planning before attempting this kind of procedure, as operator experience and volume are not always sufficient to obtain optimal results [3]. Emerging tools like artificial intelligence and machine learning might help physicians to unravel the best choice for patient outcomes in this scenario, however this technology is relatively new and larger studies are needed for this practice to become a standard in healthcare.

Percutaneous mechanical circulatory support devices (pMCS) have been often used to assist PCI in high-risk patients, especially with poor ejection fraction (EF), despite the lack of confirmative data. The first device to be widely used was the intra-aortic balloon pump (IABP) but its routine use was downgraded following the result of the IABP-SHOCK II trial in cardiogenic shock [4]. On the other hand, the Impella microaxial flow pump is a pMCS that directly increases cardiac output and unloads the left ventricle. Despite the neutral effect in small studies, retrospective analysis from recent multicenter registries suggests an increased survival in this setting [5] that might be explained by the timing of pMCS placement, as reported in a recent meta-analysis [6]. At the same time, its use is increasing in non-emergent CHIP. In this clinical setting, the effects of transient coronary occlusion due to balloon inflation and stent implantation may cause temporary myocardial hypoperfusion and hemodynamic instability, ultimately resulting in worse procedural results and long-term outcomes due to incomplete or suboptimal revascularization [7]. Nevertheless, despite the lack of consistent RCTs data, pMSC remains the most promising tool in this scenario.

The presence of multivessel disease (MVD) is often one of the main issues related to the concept of CHIP. Whereas ischemia guidance for PCI in MVD has reached a certain level of consensus in stable coronary artery disease [8] the same is often debated for patients presenting with acute coronary syndromes with MVD. Complete revascularization has been found superior to the treatment of the culprit lesion alone in patients with ST-elevation myocardial infarction STEMI [9]. However, whether complete revascularization should be guided by physiology, imaging, or angiography alone is still questioned. Recently, fractional flow reserve (FFR) has shown no significant benefits compared with an angiography-guided strategy in the management of non-culprit lesions in STEMI regarding the risk of death, myocardial infarction, or urgent revascularization at 1 year [10]. This might be since ischemia-driven revascularization in this setting may not be a rewarding strategy. A pressure wire does not seem to have sufficient capacity to discriminate the vulnerability of non-culprit atheroma while mostly defining ischemia; the latter is, in the end, a surrogate of a more complex disease such as atherosclerosis. In this clinical background, an imaging-based strategy could be a reasonable and effective solution for evaluating plaque vulnerability of non-culprit lesions and for guiding intervention as properly designed RCTs are warranted. On these grounds, optical coherence tomography (OCT) represents a useful tool to address this question as it was recently demonstrated to be a safe and feasible option to guide stenting in STEMI, with early infarct artery patency compared with angiography [11]. The “one-size-fits-all” approach might not be the best choice in MVD revascularization, especially in acute coronary syndromes. OCT, with its very high resolution, may allow accurate assessment of atheroma to guide personalized solutions for the patients [12,13].

A tailored approach to ischemia-reperfusion injury is needed. The scarcity of new therapeutic options to prevent reperfusion injury reflects the complexity of this process. In the last decades, several different therapies have been studied, but a clear effective strategy is yet to be reported [14]. From a pharmacological point of view, metoprolol and adenosine reported a reduction in the infarct size in small studies but their application failed to take root in clinical guidelines. From an interventional point of view, left ventricular unloading may represent the most innovative solution to tackle ischemia while relieving the myocardium directly from inside the ventricle during STEMI. Evidence of feasibility and safety was provided by the Door-To-Unload in STEMI Pilot Trial, involving patients with anterior STEMI without shock and randomized to Impella; this was followed by immediate reperfusion versus delayed reperfusion after 30 minutes of unloading with the microaxial flow pump [15]. Nonetheless, larger studies are needed to endorse a shift towards a “door-to-unloading” paradigm as the role of primary PCI and the “door-to-balloon” metric is yet to be challenged. Ultimately, a large thrombus burden is often a jeopardizing situation in complex anatomies that might be the cause of no-reflow/slow-flow and incomplete recanalization of infarct-related arteries. In this regard, new devices such as the latest mechanical thrombectomy catheter “NeVa” [16] have demonstrated excellent efficacy and should be tested in larger RCTs.

Lastly, there is increasing evidence that new-generation drug-eluting stents (DESs) with thinner struts are associated with better outcomes [17]. Reduction of strut thickness has been an important innovation in stent design, leading to easier manipulation, reduced risk of stent thrombosis, and lower rates of revascularization. Noteworthy, the largest randomized controlled trial in this area, the BIORESORT [18] demonstrated that struts ranging from 60 to 81 μm showed excellent outcomes in terms of target vessel failure rates. However, less than 30% of enrolled patients had bifurcation PCI and less than 2% unprotected left main. On this point, thin and ultra-thin DESs appear very attractive in treating complex lesions, especially in small vessels and bifurcations but available data is scarce and mostly comes from retrospective analysis [19].

Complex coronary intervention is more and more becoming a tailoring art. Complexity dwells either in the presenting anatomy, in the knowledge of technologies, or the clinical history of the patient. Frequently, the treating physician, beyond appropriately balancing the risk and benefits of interventions, is forced to guarantee excellent results while facing a growing demand for minimally invasive treatments. In this optic Machine Learning (ML) artificial intelligence has delivered impressive results, and we expect its application in this setting to increase in the next few years [20]. One question bears asking: are we heading in the right direction?
